# Critical Care Education Day: A Novel, Multidisciplinary, and Interactive Critical Care Education Session for Emergency Medicine Residents

**DOI:** 10.7759/cureus.6785

**Published:** 2020-01-27

**Authors:** Natalie N Htet, Alexandra June Gordon, Tsuyoshi Mitarai

**Affiliations:** 1 Emergency Medicine, Stanford University Medical Center, Palo Alto, USA

**Keywords:** critical care education, emergency medicine education, medical education, simulation, multidisciplinary, residency, small group sessions, non lecture format

## Abstract

Critical care medicine (CCM) is central to emergency medicine (EM) resident education. We feel that the traditional lecture format is not the ideal way to teach EM critical care, which requires integration and prioritization of diagnostic workup and team-based resuscitation under time pressure. We describe a novel critical care education day where an interactive, practical, and multidisciplinary critical care educational experience was provided for EM residents using case-based small-group sessions and fast-paced simulation.

## Introduction

CCM is integral to EM residency education, yet it is difficult to take time to teach at the bedside as the resuscitation of patients requires close attention and time-sensitive interventions [1,2,3]. In addition, learning styles vary among EM residents, and the clinical setting of critical care makes it difficult to cater to all learners [4]. However, the classroom teaching format has limitations in that it does not train the audience to integrate cognitive and procedural tasks under time constraints [5]. Furthermore, imparting knowledge of complex critical care topics to junior residents without adequate exposure to direct patient care may hinder their ability to properly absorb the information [6-9].

In order to improve critical care education, a multidisciplinary critical care education day for EM residents was created during their weekly conference time at a single, urban, academic EM program. This report will serve to guide residency directors and educators in EM to incorporate multidisciplinary, interactive, and fast-paced sessions in critical care education.

## Technical report

Schedule

The learners began the day with a 30-minute series of rapid-fire questions and answers that were based on the learning objectives for the day. This was intended to spark curiosity in learners and to introduce the breadth of topics for the rest of the day. The second session was a 45-minute multidisciplinary panel discussing peri-intubation management of unstable patients. A senior EM faculty member moderated short cases with high-risk conditions, such as severe metabolic acidosis, pulmonary hypertension, hemodynamic instability, and severe hypoxemia with agitation. During the last three hours, residents were divided equally among residency classes into six groups and rotated through six small-group critical care stations for 25 minutes each (Table 1).

**Table 1:** Schedule of the critical care day and objectives of each station ED: emergency department; EM: emergency medicine; RV: right ventricle; PEEP: positive end-expiratory pressure; IVC: inferior vena cava

Time	Station title	Objectives and Contents	Instructor
7:30 AM–8 AM	Rapid-fire critical care-related questions and answers game	Introduce the breath of critical care topics residents will encounter during the day	EM-trained critical care faculty and fellows
8 AM–8:55 AM	Panel: peri-intubation strategy for critically ill patients	Discuss various techniques on optimizing hemodynamic status and preventing a peri-intubation arrest of patients with severe metabolic acidosis, refractory hypoxemia with agitation, shock, pulmonary hypertension, and RV failure	Panel of EM faculty, anesthesia critical care faculty, cardiac anesthesia critical care faculty, and internal medicine/anesthesia critical care faculty
9 AM-12 PM	Small-group stations		
25 minute-sessions per station	1) Cardiac arrest	Provide advanced training in post-cardiac arrest care: sedation, ventilator, and ionotropic support. Recognize ST-elevation myocardial infarction, and activate the cath lab. Consider extracorporeal membrane oxygenation	EM-trained critical care fellow and simulation faculty
	2) Ventilator troubleshooting and experiencing positive pressure ventilation	Experience positive pressure ventilation. Provide a differential diagnosis for high-peak pressures with low-plateau pressures vs. high-plateau pressures. Demonstrate understanding of permissive hypercapnia in an acute asthma presentation. Describe the role of PEEP for hypoxemic asthma patients	EM-trained critical care fellow
	3) Blood gas analysis	Discuss common acid-base problems: acute vs. chronic respiratory acidosis and metabolic compensation, Winter’s formula, hyperchloremic metabolic acidosis, and delta gap	EM-trained critical care fellow
	4) Neuro-resuscitation in the ED	Describe grading scales used to assess patients with neurologic injury (specifically acute stroke and traumatic brain injury) and discuss when and why scales should be used. Discuss the rationale and indications for neurosurgical intervention in acute intracerebral hemorrhage and traumatic brain injury. Discuss tiered management of intracranial hypertension and the risks-benefits to therapies	Neurointensivist
	5) Undifferentiated shock	Recognize the importance of early and serial use of focused cardiac ultrasound and lung ultrasound in patients with undifferentiated shock. Categorize patients with shock into cardiogenic, obstructive, hypovolemic, or distributive shock by integrating clinical picture, IVC, focused cardiac ultrasound, and lung ultrasound. Recognize the dynamic nature of shock and adjust the management strategy accordingly	Simulation faculty; EM-trained critical care faculty and fellows
	6) Pediatric critical care	Appreciate the physiology of pediatric patients with sickle cell disease. Differentiate aplastic crisis from other acute causes of anemia in patients with sickle cell disease. Understand the management of aplastic crisis	Pediatric EM faculty/pediatric intensivist

Settings

Five rooms in a simulation center were reserved for the day. A large room with a flexible desk and chair arrangement was used for the rapid-fire session, expert panel discussion, and neurocritical care and acid-base stations. For the rest of the sessions, one large simulation suite and three smaller rooms were used. The case of shock was conducted in a simulation suite that was outfitted to be similar to an emergency department (ED). A hospital-type room with a mannequin and a ventilator was set up for the ventilator-associated teaching station. For the cardiac arrest station, a crash cart, a mannequin with an adult male upper torso, and a monitor that displayed vital signs were used.

Participants

Forty residents and eight medical students participated in the critical care education day. Educators from various disciplines were asked to participate and to provide specific training. These included physicians from cardiac anesthesia, neurocritical care, EM, and adult and pediatric intensive care units (ICU); EM-trained critical care fellows; respiratory therapy education specialists; pharmacists; and simulation team.

Small-group stations

1) Ventilator Management

This case was designed to allow learners to apply positive pressure ventilation on themselves and to teach peak vs. plateau pressure. Using an individual mouthpiece, learners experienced what it was like to breathe on a ventilator for a few minutes with various ventilator settings. Afterward, learners ran through a simulation case as a group.

Case: a 58-year-old man with severe asthma was brought in by paramedics for shortness of breath. The patient was intubated for respiratory distress.

Learners encountered a situation where the ventilator was alarming and the patient’s pulse oxygen saturation was decreasing. Learners had to listen for bilateral breath sounds to assess for pneumothorax, recognize high auto-positive end-expiratory pressure (auto-PEEP), disconnect the ventilator, and adjust the respiratory rate and inspiratory to expiratory ratio to allow adequate time for exhalation. The case was concluded with a debriefing session on the importance of recognition of auto-PEEP and the use of permissive hypercapnia in patients with obstructive lung disease and to recognize the findings of auto-PEEPing on a ventilator [10,11].

2) Cardiac Arrest

This case was designed to provide an overview of the bundle of care involved in the care of a post-cardiac arrest patient, rather than re-demonstrate standard advanced cardiac life support (ACLS) management. 

Case: a 60-year-old woman in cardiac arrest was brought in by ambulance to the ED.

Paramedics found the patient on the street and no further history was available. The patient was in a shockable rhythm and she got a return of spontaneous circulation (ROSC) immediately after defibrillation.

The instructor focussed on minimizing interruptions in chest compressions by continuing cardiopulmonary resuscitation (CPR) until the defibrillator was fully charged. Learners were directed to focus on post-arrest care by the instructor. The case was completed when learners obtained labs and imaging to diagnose the cause of arrest, managed sedation and blood pressure, and initiated hypothermia post-ROSC [12].

3) Acid-base

The acid-base station consisted of three cases that were run around a table.

Case 1: a 68-year-old woman with severe chronic obstructive pulmonary disease (COPD) presented to the ED with shortness of breath. Her blood gas demonstrated a pH of 7.21, pCO2 of 81 mmHg, and pO2 of 63 mmHg. Her basic metabolic panel was as follows - sodium: 138 mEq/L, potassium: 3.8 mEq/L, chloride: 97 mEq/L, bicarbonate: 32 mEq/L, blood urea nitrogen (BUN): 22 mg/dL, creatinine: 0.9 mEq/L, and glucose: 166 mg/dL.

Learners had to identify whether the patient had an acute respiratory acidosis vs. an acute on chronic respiratory acidosis [13].

Case 2: a 37-year-old man with diabetes presented with shortness of breath. His arterial blood gas showed a pH of 7.02, pCO2 of 10 mmHg, and pO2 of 72 mmHg. His finger stick glucose was over 600 mg/dL. After starting an insulin drip and fluid, the repeat metabolic panel was as follows - sodium: 144 mEq/L, potassium: 3.8 mEq/L, chloride: 123 mEq/L, bicarbonate: 15 mEq/L, BUN: 17 mg/dL, creatinine: 0.6 mEq/L, and glucose: 266 mg/dL.

Learners had to use Winter’s formula to understand whether the patient was appropriately compensating for metabolic acidosis. Learners also learned that hyperchloremia from normal saline could cause a non-anion gap metabolic acidosis [13].

Case 3: a 51-year-old man with chronic alcohol usage presented to the ED with vomiting and abdominal pain. ABG revealed a pH of 7.40, pCO2 of 41 mmHg, pO2 of 85 mmHg, and HCO3 of 22 mEq/L. His metabolic panel was as follows - sodium: 137 mEq/L, potassium: 3.8 mEq/L, chloride: 90 mEq/L, bicarbonate: 22 mEq/L, BUN: 36 mg/dL, creatinine: 1.7 mEq/L, and glucose: 96 mg/dL.

Learners had to calculate the anion gap and the delta gap to appreciate that the patient had a mixed metabolic alkalosis and metabolic acidosis. Learners were taught the causes of anion gap metabolic acidosis, including toxic alcohol ingestions [13].

4) Neurocritical Care

The neurocritical care station consisted of a case taught at a round table. The case involved an emergency medical services (EMS) ring-down and multiple CT stimuli.

Case: a 55-year-old woman collapsed while watching a sporting event. She was found to have an intracerebral hemorrhage.

Learners were instructed on EM of neurological injury, the importance of oxygenation and ventilation, head of the bed position, sedation, blood pressure management, and indications for neurosurgical intervention [14].

5) Undifferentiated Shock

Case: a 60-year-old man with acute leukemia status post-chemotherapy one week prior arrived at the ED with fever, tachycardia, and hypotension. Initial vitals were as follows: heart rate: 130, blood pressure: 76/38, respiratory rate: 24, temperature: 38.6 °C, and SpO2: 97%. Labs are described in Table 2. Additional materials included an electrocardiogram (EKG) with sinus tachycardia and a normal chest X-ray. If learners requested an echo, a video of an echo was shown (good contractility, though hyperdynamic; no pericardial effusion and no obvious right heart strain). They were also shown an inferior vena cava (IVC) ultrasound with respiratory variation and a lung ultrasound with A-lines and no B-lines. The PowerPoint (Microsoft, Redmond, WA) was used for both medical information ( video clips, EKG, and labs) as well as teaching points, and it was projected onto a large screen near the mannequin.

**Table 2:** Labs for the undifferentiated shock station BUN: blood urea nitrogen

Complete blood count	Basic metabolic panel	Venous blood gas
White blood cell: 0.1 x 10^3^/microL	Sodium: 129 mEq/L	pH: 7.15
Hemoglobin: 7.3 mmol/dL	Potassium: 3.7 mEq/L	pCO_2_: 26 mmHg
Hematocrit: 22 mmol/dL	Chloride: 91 mmol/L	Lactate: 6
Platelet: 32x 10^3^/microL	CO_2_: 19 mmol/L	
	BUN: 46 mg/dL	
	Creatinine: 1.8 mEq/L	
	Glucose: 178 mg/dL	

**Figure 1:**
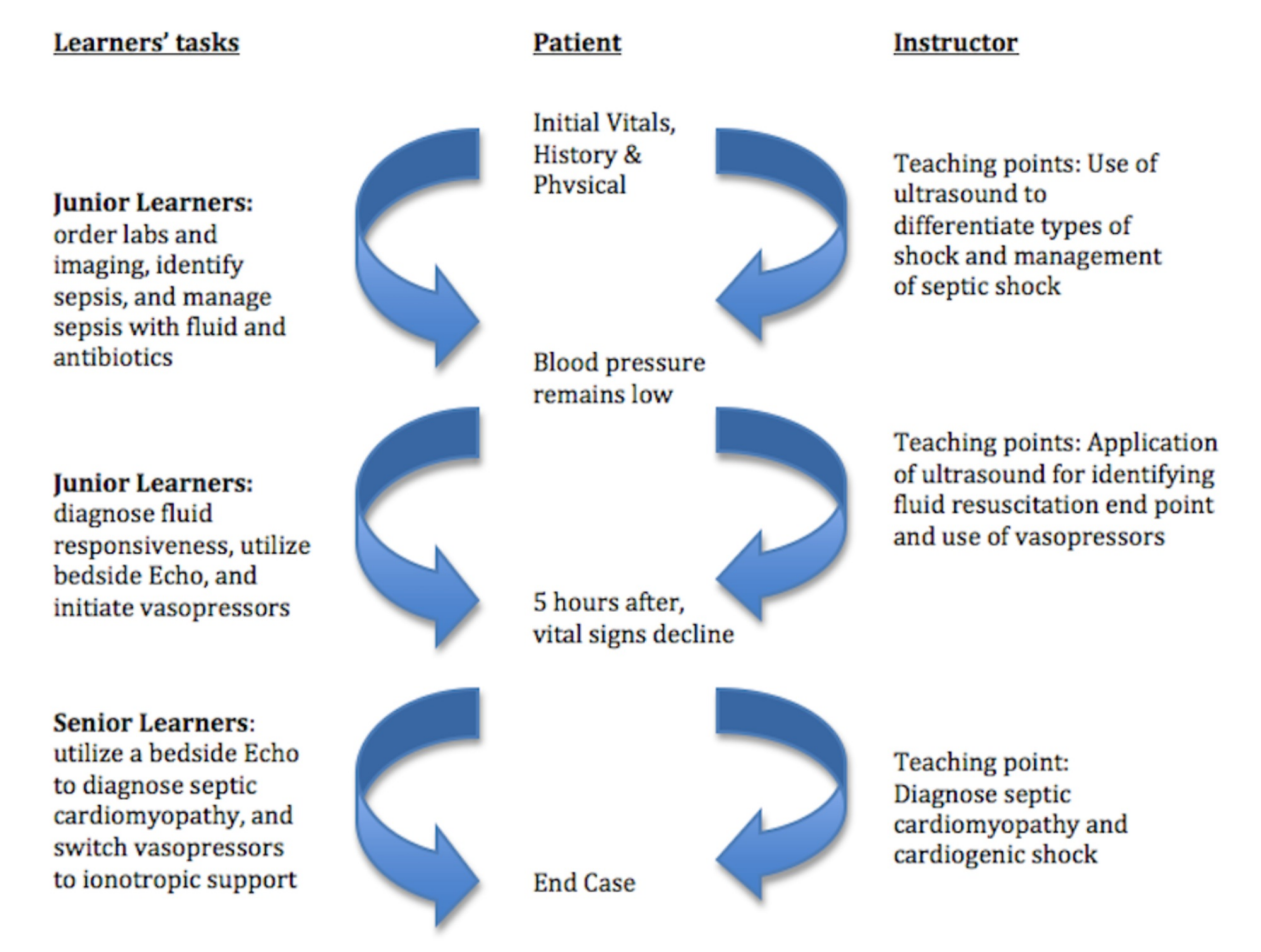
Flow chart with branch points for undifferentiated shock simulation station Junior learners: first-year residents paired with second- or third-year residents; senior learners: third-year residents paired with second-year residents

The scenario was broken into three phases. Residents were divided into three groups of junior and senior learners, each consisting of 2-3 residents. In the junior learners' group, first-year residents (R1) were paired with second or third-year residents (R2, R3) to tackle the first and second phases of the simulation. In the senior learners' group, R3s were paired with R2s to solve the last phase of the simulation. Simulation with two or more participants required collaboration and vocalization of the learner’s thought processes, and it was ensured that everyone was able to participate in the allotted time. Furthermore, each resident was instructed to function at a level one year above their given year of training to make it more challenging. Each phase had pre-selected tasks that the learners had to compulsorily complete and a “time out” was called when the majority of tasks were completed. These timeouts allowed for a smooth and controlled flow of the session and timely teaching for each phase, which helped the learners to solidify the information (Figure 1).

In phase 1, junior learners had to identify and manage sepsis including early antibiotics and 30mL/kg of fluid resuscitation. In phase 2, despite such intervention, blood pressure remained low, and the junior learners were tasked to utilize appropriate vasopressors and figure out the endpoint of fluid resuscitation using focus cardiac and lung ultrasound [15]. In phase 3, the patient was admitted to ICU, but no bed was available. The case was fast-forwarded to five hours later, where the patient deteriorated and was administered maximum doses of norepinephrine and vasopressin with a concurrent rise in lactate level. The case ended if senior learners could identify newly developed septic cardiomyopathy with cardiogenic shock and replaced the vasopressors with inotropic agents [15].

6) Pediatric Resuscitation

The pediatric resuscitation station involved a low-fidelity mannequin in a small classroom.

Case: A 12-year-old boy presented with leg pain, tachycardia, and tachypnea. Although the patient appeared to be having a pain crisis initially, his hemoglobin was below his baseline and he had a low reticulocyte count.

Learners had to resuscitate a patient with sickle cell disease in an aplastic crisis. Learners were taught the pathophysiology of children with sickle cell disease and the management of aplastic crisis with blood transfusion in pediatric patients [16].

## Discussion

Graduate medical education in EM is largely focused on weekly lectures [17]. In our institution, critical care has been taught mainly through emergency medicine critical care medicine (EMCCM) joint case conference series, offered every 1-2 months for several years. Despite its popularity, it is not an ideal format to teach resuscitation, where the nuances can mean life or death, and the stress and stakes are the highest [2]. We felt that a fast-paced simulation and interactive small-group sessions would serve as an ideal supplement to the existing critical care curriculum to teach teamwork, decision making under time pressure, and resuscitation with real-time feedback [18]. In this report, we described the creation and implementation of a critical care education day consisting of multidisciplinary, interactive, fast paced education sessions in a non-lecture format. The critical care education day received the year's highest didactic ratings by the residents, and it is currently offered on an annual basis with adjustments of themes and scenarios based on feedback and requests from the residents.

The concept was implemented successfully and provides a practical insight for other institutions that wish to offer a critical care education day. Even if an institution does not have any EMCCM physicians, collaboration with faculty and fellows both within and outside of EM specialties would make such an event possible. In fact, we believe a variety of perspectives and skill sets brought by multidisciplinary educators was central to the success of the critical care day; and we recommend such collaborations not only with physicians, but also with nurses, respiratory therapists, and pharmacists. The planning should start several months in advance to secure the appropriate space for the event and ensure sufficient time for preparation. Themes for each small group should be selected first, based on the input from the residents and fellows volunteering to be part of the event planning committee. Once the themes are determined, ideal instructors for the corresponding themes can be identified from within the institution. Lastly, the instructor for each small group should be paired up with residents from the committee to ensure that the learners' perspectives are reflected in the learning objectives and case selection.

Lessons learned

During the simulation, the pairing of two or more participants was found to be critical for two reasons: it ensured that everyone got to participate in the allotted time and it forced collaboration and vocalization of the learner’s thought processes. Giving upgraded roles for the residents during the simulation (i.e., R1 acts as R2; R3 acts as attending) is also recommended for an additional educational experience. Liberal use of “fast forward” for procedures and test results is highly recommended to keep the session efficient and result-oriented. Lastly, rather than providing all debriefings at the end, we recommend the use of “time outs” to provide teaching points at the end of each phase of simulation while the case is still fresh in the learners' minds.

The allocated time of 25 minutes per session was not long enough, and it was increased to 35 minutes per session with up to three learning objectives in the subsequent years. In addition, we eliminated the rapid-fire session and panel discussion to gain extra time for the small- group sessions but added a Jeopardy-style session during lunch to reinforce teaching points from each station.

Limitations

Evaluating a new learning method with traditional teaching methods is difficult in graduate medical education, as the medical education community still needs to address several major challenges in the assessment of trainees in competency-based medical education [19]. Although we compared the learners' review of the critical care day with traditional lectures at our institution, the analysis had to have several confounders to be included in the paper. We did not measure the learners' confidence or self-perceived competence in procedural or cognitive domains, which contrasted with other papers [3,7]. We initiated the project as a proof of concept to improve critical care teaching to residents, and we plan to put in place a more robust method of analysis in the upcoming years.

## Conclusions

In this paper, we introduced a novel concept: a critical care education day consisting of multidisciplinary, interactive, fast-paced critical care education sessions in a non-lecture format for EM residents. We recommend incorporating such sessions to the existing critical care curriculum in other EM residency programs to teach teamwork, decision making under time pressure, and resuscitation with real-time feedback.
